# *Helicobacter pylori* Infection and Peptic Ulcer Disease in Symptomatic Children in Southern Vietnam: A Prospective Multicenter Study

**DOI:** 10.3390/healthcare11111658

**Published:** 2023-06-05

**Authors:** Tu Cam Nguyen, Ngoc Le Chau Tang, Giao Kim Ngoc Le, Vy Thuy Nguyen, Khuong Hoang Gia Nguyen, Thai Hoang Che, Van Thi Tuong Phan, Ngoc Minh Nguyen, Dinh Quang Truong, Xuan Minh Ngo, Hiep Thanh Nguyen, Annie Robert, Patrick Bontems, Phuong Ngoc Van Nguyen

**Affiliations:** 1 Department of Gastroenterology, City Children’s Hospital, Ho Chi Minh City 700000, Vietnam; 2 Faculty of Medicine, Université Libre de Bruxelles, 1020 Brussels, Belgium; 3 Department of Gastroenterology, Children’s Hospital 2, Ho Chi Minh City 700000, Vietnam; 4 Department of Microbiology and Parasitology, University of Medicine and Pharmacy, Ho Chi Minh City 700000, Vietnam; 5 Department of Genetics, University of Science—Vietnam National University Ho Chi Minh City, Ho Chi Minh City 700000, Vietnam; 6 Department of Biostatistics and Informatics, Pham Ngoc Thach University of Medicine, Ho Chi Minh City 700000, Vietnam; 7 Department of Surgery, City Children’s Hospital, Ho Chi Minh City 700000, Vietnam; 8 Faculty of Medicine, Pham Ngoc Thach University of Medicine, Ho Chi Minh City 700000, Vietnam; 9 Faculty of Public Health, Pham Ngoc Thach University of Medicine, Ho Chi Minh City 700000, Vietnam; 10 Institut de Recherche Expérimentale et Clinique, Pôle d’Épidémiologie et Biostatistique, Université Catholique de Louvain, 1200 Brussels, Belgium; 11 Department of Gastroenterology, Hôpital Universitaire des Enfants Reine Fabiola, 1020 Brussels, Belgium

**Keywords:** *Helicobacter pylori*, peptic ulcer disease, symptomatic children, virulence factors, biopsy-based tests

## Abstract

Background: *Helicobacter pylori* (*H. pylori*) remains a major cause of gastroduodenal diseases. We aimed to evaluate the burden of this infection, particularly peptic ulcer disease in Vietnamese children. Methods: We enrolled consecutive children referred for esophagogastroduodenoscopy at two tertiary children’s hospitals in Ho Chi Minh City, from October 2019 to May 2021. Children treated with proton pump inhibitors during the last two weeks or antibiotics for four weeks, and those having a previous or interventional endoscopy were excluded. *H. pylori* infection was diagnosed with either a positive culture or positive histopathology combined with a rapid urease test, or with a polymerase chain reaction of the urease gene. The study was approved by the Ethics Committee and written informed consent/assent was obtained. Results: Among 336 enrolled children aged 4–16 (mean: 9.1 ± 2.4 years; 55.4% girls), *H. pylori* infection was positive in 80%. Peptic ulcers were detected in 65 (19%), increasing with age, and 25% with anemia. *cagA+* strains were detected at a higher rate in children with ulcers. Conclusions: Prevalence of *H. pylori* and peptic ulcers is high among symptomatic Vietnamese children. It is crucial to have a program for early detection of *H. pylori* to reduce ulcer risk and gastric cancer later.

## 1. Introduction

*Helicobacter pylori* (*H. pylori*) infection remains a significant cause of human gastroduodenal diseases, especially in highly prevalent countries. It is commonly acquired during childhood and can have long-term consequences. *H. pylori* may cause chronic gastritis and gastroduodenal ulcers, affecting both children and adults [1,2]. It can sometimes progress to stomach cancer decades later [3]. Although rare, *H. pylori* infection can also lead to gastric mucosa-associated lymphoid tissue lymphoma, particularly in children [4]. Furthermore, it may be associated with certain extra-intestinal diseases such as chronic idiopathic thrombocytopenic purpura, iron deficiency anemia, Henoch–Schonlein purpura, and nutritional status [5].

Its clinical manifestations are diverse, nonspecific, and can be asymptomatic. Diagnosis of *H. pylori* infection in children relies on an upper gastrointestinal (GI) endoscopy using biopsy-based diagnostic tests [6], with acceptable indications for upper GI endoscopies described by Tringali et al. [7].

Vietnam is regarded as an emerging country, but *H. pylori* prevalence in the community remains high [8,9]. *H. pylori* infection was estimated at 70.3% of the Vietnamese population in 2017 [8]. In 2022, the prevalence of *H. pylori* was reported at 87.7% in school-aged children in Ho Chi Minh City [10]. 

Clinical data in symptomatic, scoped, Vietnamese children are rare. Recent clinical series have reported an increase in *H. pylori*-induced gastritis and peptic ulcer disease (PUD) among children over the past decade [11,12,13,14]. However, comparing across centers is difficult because diagnostic tests depend upon local resources, not always aligned with international standards. Therefore, extensive hospital-based, prospective, multicenter studies are needed to determine the infection rate, clinical features, and endoscopic findings of *H. pylori* infection in symptomatic Vietnamese children. We conducted such a study in two pediatric reference endoscopic facilities in Ho Chi Minh City, Vietnam’s largest city.

## 2. Materials and Methods

### 2.1. Study Population and Design

The study was conducted at two pediatric hospitals in Ho Chi Minh City (HCMC), from October 2019 to May 2021. The 1400-bed Children’s Hospital 2 and the 1000-bed City Children’s Hospital serve as primary facilities for the local population in HCMC and as the tertiary referral centers for children from 18 southern provinces, with a catchment population of over 2.4 million children [15]. We recruited consecutive children aged 4 to 16 years assigned to upper GI endoscopy due to alarm symptoms according to the guidelines of the European Society of Gastrointestinal Endoscopy (ESGE) and European Society for Pediatric Gastroenterology Hepatology and Nutrition (ESPGHAN) [6]. Alarm symptoms include persistent right upper quadrant pain, dysphagia, odynophagia, persistent vomiting, GI blood loss, and weight loss. Children underwent therapeutic endoscopic procedures; those with a previous history of upper GI endoscopy; those who had taken proton-pump inhibitors (PPIs), 2-histamine receptor antagonists, antacids, and bismuth salts within two weeks, or antibiotics within four weeks before endoscopy, were excluded from the study. Data on demographic characteristics, history of *H. pylori* infection, underlying diseases, clinical symptoms, and endoscopic findings were collected. Nutritional status was determined using anthropometric measurements and classified as underweight, normal weight, overweight, and obesity according to the World Health Organization Child Growth Standards Chart.

Written informed consent of parents/legal guardians was obtained before data and sample collection. Children older than 12 years also signed informed assent. The study was approved by the Scientific Council of the Pham Ngoc Thach University of Medicine (No 2683/QĐ-TĐHYKPNT) and the local Ethics Committees of two hospitals (No 37/QĐ-BVNĐTP).

### 2.2. Gastrointestinal Endoscopy and H. pylori Tests

Each patient underwent an upper GI endoscopy, with macroscopic findings recorded for the esophagus, gastric corpus, gastric antrum, and duodenum. Endoscopic lesions were categorized based on the most severe lesion in ascending order of erythema, nodularity, erosion, and ulceration if both gastric and duodenal lesions were present, using Minimal Standard Terminology for Digestive Endoscopy [16]. The Los Angeles Classification [17] was used for grading erosive esophagitis. The Forrest classification was used for peptic ulcers with spurting hemorrhage (Forrest Ia), oozing hemorrhage (Forrest Ib), non-bleeding visible vessel (Forrest IIa), adherent clotting (Forrest IIb), flat pigmented hematin on an ulcer base (Forrest IIc), and a clean base ulcer (Forrest III) [18]. Following ESPGHAN recommendations [7], three antrum and two corpus biopsy specimens were taken routinely, whenever not contra-indicated, for *H. pylori* culture, histopathology, rapid urease test (RUT), and polymerase chain reaction (PCR) assay of urease gene-*ureA*, cytotoxin-associated gene A (*cagA*) gene, and vacuolating cytotoxin A (*vacA*) genotypes during the endoscopy. 

Two biopsies (one antrum, one corpus) for *H. pylori* culture were preserved separately in specialized carrying media, brain heart infusion broth, with 1% agar at 4 °C. They were transported within 4 h to the laboratory. Plates were incubated for up to 14 days at 35 °C under microaerophilic conditions. Positive reactions for oxidase, catalase, and urease identified isolates of *H. pylori*. 

Two other biopsies (one antrum, one corpus) were preserved in a 10% formalin solution and stained with Hematoxylin-Eosin and Giemsa. Histological classification was defined using the updated Sydney classification system [19].

The remaining gastric antrum biopsy was used for on-site RUT (Urease NS, Vieta Corporation, Ho Chi Minh City, Vietnam) and PCR assays for the urease gene-ureA, as well as virulence factors, such as *cagA* gene and *vacA* genotypes. Real-time PCR was performed using the AccuPid *H. pylori* Detection Kit (KT Biotech, Ho Chi Minh City, Vietnam) for urease gene detection. Urease PCR-positive samples were further genotyped for *cagA* and *vacA* genes using Multiplex PCR with the AccuLite *H. pylori* Genotyping Kit (KT Biotech, Ho Chi Minh City, Vietnam) and primer sequences from a previous Vietnamese study [20]. After that, they were then synthesized using Integrated Device Technology. Multiplex PCR was performed in Biometra (Tprofessional Thermocycler Family). PCR products for each reaction were electrophoresed on 5% agarose gel, stained with ethidium bromide, and visualized under UV light. 

The diagnosis of *H. pylori* infection was based on either a positive culture or the combination of histological examination with at least one other positive biopsy-based test (RUT or PCR), following ESPGHAN/NASPGHAN criteria [6]. *H. pylori* status was defined as doubtful if the culture was negative with one other positive biopsy test.

### 2.3. Statistical Analysis

Data were coded and recorded using EpiData software version 4.6.0 (EpiData Association, Odense, Denmark), with encoding performed by two data managers. Data monitoring was conducted by the first author (T.C.N.), and additional plausibility checks were performed before exporting to StataSE 17.0 (Stata Corporation, College Station, TX, USA) for analysis. Initially, a descriptive analysis was performed to determine the *H. pylori* infection rate. Subsequently, a univariable analysis was performed using the chi-square test (or Fisher’s exact test) to compare *H. pylori* infection between the categories of variables (gender, living area, history of gastroduodenal disease, history of *H. pylori* infection, nutritional status, clinical manifestations, endoscopic findings, *cagA* status, and *vacA* genotypes). For ordered categorical variables such as age subgroups, the Cochran–Armitage test for trend was used. In the multivariable logistic regression model, variables were entered if they had a univariate *p*-value < 0.25. This model was built for identifying factors independently associated with *H. pylori* infection or with PUD. A *p*-value less than 0.05 was considered significant.

## 3. Results

### 3.1. Patient Characteristics

**Figure 1:**
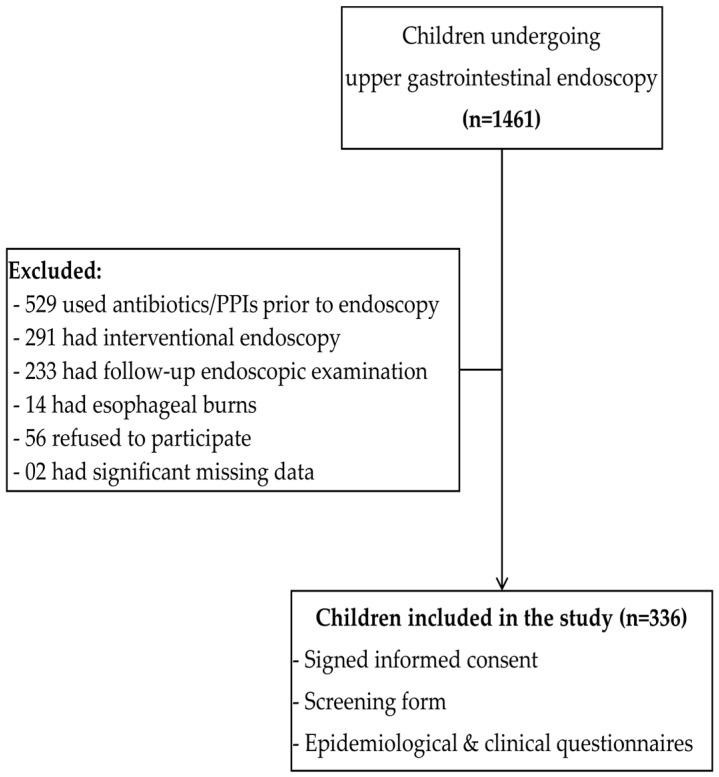
Flowchart of the study series.

From October 2019 to May 2021, among 1461 consecutive children undergoing upper GI endoscopy at the two children’s hospitals in HCMC, 336 patients who met the criteria were included in the study (Figure 1).

The mean age was 9.1 ± 2.4 years (range: 4.2–15.3 years) and 55.4% (186/336) were girls. The proportion of boys increased significantly with age: 33.3% (8/24) were in the group under 6 years, 40.8% (97/238) in the group aged 6–11 years, and 60.8% (45/74) in the group above 11 years. Most children lived in HCMC (39.9%), followed by South-East provinces (28%), Mekong River Delta (22.9%), and other regions (9.2%). Children presented with an average of four GI symptoms at endoscopy. The most common symptoms were chronic abdominal pain (95.2%; 320/336) and epigastric pain (75.3%; 253/336). More than a third of patients showed accompanying symptoms (vomiting, constipation, regurgitation, weight loss, and loss of appetite).

### 3.2. H. pylori Infection Rate in Symptomatic Children

**Figure 2:**
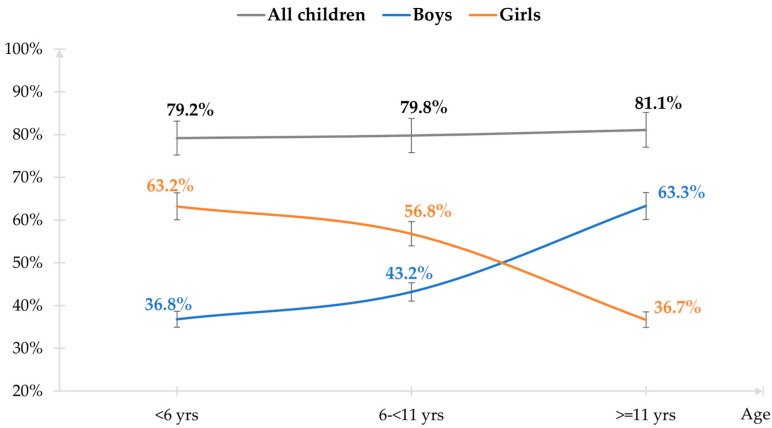
Distribution of *H. pylori* infection according to age group and gender.

The positivity rate was 39.0% (131/336) for *H. pylori* culture, 79.7% (267/335) for histopathology, 83.6% (280/335) for PCR ureA, and 83.6% (281/335) for RUT. *H. pylori* status was confirmed positive in 80.1% of patients (269/336), doubtful in 9.2% (31/336), and negative in 10.7% (36/336). The prevalence of *H. pylori* infection was similar between age groups and gender but varied between boys and girls across different age groups (Figure 2). 

Living in HCMC was associated with higher *H. pylori* infection rates in both univariable and multivariable logistic regression (OR = 4.28; 95% CI: [2.15–8.55]; *p* < 0.001), while there was no difference between urban and rural residents of HCMC. 

No association was found between demographic characteristics, history of gastroduodenal diseases, history of *H. pylori* infection, nutritional status, clinical symptoms, and *H. pylori* infection (Table 1). 

### 3.3. H. pylori Infection and Gastroduodenal Endoscopic Lesions

Among 336 symptomatic children, the most common mucosal abnormalities were nodularity, accounting for 60.7% (204/336), followed by ulcers (19.4%, 65/336), erythema (12.2%; 41/336) and erosion (7.7%; 26/336). Nodularity was commonly observed in the stomach. Ulcers were predominantly located in the duodenal bulb (93.9%, 61/65), both gastric and duodenal ulcers (4.6%, 3/65), and in the stomach (1.5%,1/65). Erosive esophagitis was reported in only 1.8% of the children (6/336) (Table 2). 

Regarding peptic ulcers, the median diameter was 7 mm (IQR: 5–15 mm). Multiple ulcers were observed most frequently, accounting for 50.8% (33/65). Forrest classification was available in 37 out of 65 cases (Forrest class III: 91.9%; Forrest class IIb: 8.1%). Among the children with ulcers, nodular gastritis was found in 83.1% (54/65). 

*H. pylori* infection was positive in 93.8% of children with ulcers (61/65) and in 76.8% of children without ulcers (208/271). There was a significant association between nodularity, PUD, and *H. pylori* infection. Ulcers appeared more commonly in boys and with increasing age. Children with ulcers presented frequently with anemia (15/32; 46.9%) and melena (11/20; 55%), but this significant association was not observed in multiple analysis. Table 3 provides further information on demographics, family history of PUD, nutritional status, clinical symptoms, and virulence factors of *H. pylori*-positive children with and without ulcers. 

### 3.4. H. pylori Virulence Factors

PCR was successfully performed in 268 out of the 269 children diagnosed with *H. pylori* infection, and *cagA* was detected in 69% (185/268) of the cases. *H. pylori* isolates carrying the *cagA* gene had a higher risk of ulceration (OR: 3.25, (95% CI: 1.37–17.71); *p* = 0.008). *vacA* genotypes were associated with ulcers in univariable analysis, but not significantly different in the multivariable analysis. In children with ulcers, the *vacA s1/m1m2* was higher while the *vacA s1/m2* genotype was lower.

## 4. Discussion

The overall prevalence of *H. pylori* infection among symptomatic Vietnamese children was approximately 80%, much higher than that reported worldwide (21.9%) [21] and in Southeast Asia (48.4%) [21]. When confirmed and doubtful infections are pooled, the infection rate reached 89.2%. This infection rate is 1.8 to 2.3-fold higher than the rate observed in the communities in the Northern and central provinces of Vietnam such as Hanoi (34%) [22], TayNguyen (40%) [23], and HoaBinh (42.8%) [24], but it is close to the rate of 87.7% among school-aged children in HCMC [10] and of 90% among hospitalized children in Hanoi [25]. These findings indicate an alarming prevalence of *H. pylori* infection in symptomatic children in Southern Vietnam. 

Interestingly, children living in HCMC had higher *H. pylori* infections than in neighboring provinces, which supports findings from a community-based study conducted in HCMC with school-aged children from 6 to 15 years (87.7%) [10]. The overcrowded living conditions explain this finding. HCMC has a high population density of 4375 people/km^2^, in contrast to lower population densities in the South-East regions (778 persons/km^2^), and the Mekong Delta region (426 persons/km^2^) [15]. However, the infection rate was similar between urban and rural areas, which is consistent with findings in a hospital-based study in Hanoi [22], but different from a community-based study in HCMC that showed higher infection rates in urban areas [10]. In Vietnamese tradition, living together across generations, and sharing beds, dishes, and food with chopsticks may increase *H. pylori* infection risk [22,24,26,27]. Regarding socioeconomic factors, in a community-based study in HCMC using a stool antigen test, all crowding factors such as population density, employee density, family size, and the number of children in a household were significantly associated with a high prevalence of *H. pylori* infection [28]. Age and gender were not associated with *H. pylori* infection in this study, which is in line with other research [21,29,30,31]. *H. pylori* infection typically increases with age, with a prevalence ranging from 31.6% in high-income countries to 54.7% in low-income countries [21,29,32]. This may be related to our study series being selective in hospitals while the community-based studies used random sampling and as such, are more representative. However, *H. pylori* transmission appeared to occur at an early age, as 79.2% of children between 4 and 5 years of age were already infected.

Children with *H. pylori* may exhibit GI symptoms or may only be diagnosed when complications occur, such as upper GI bleeding or perforation from peptic ulcers [1,2]. The main indications for upper GI endoscopy in our study were persistent abdominal pain (95.2%) and epigastric pain (75.3%). Although the association between clinical symptoms and *H. pylori* infection was not significant, these findings highlight the importance of clinicians considering *H. pylori* gastritis and PUD as potential organic causes in children with alarming GI symptoms.

Regarding endoscopic lesions, most patients had abnormal appearances in the stomach and duodenum. Despite all children being symptomatic and referred for endoscopy due to suspected organic causes, the diagnostic yield was remarkably high. The most common endoscopic lesions in children were nodularity (60.7%), of which 83.8% were positive for *H. pylori*. Nodularity has been reported in infected children, ranging from 21 to 93% [11,13,33,34,35,36]. It is a typical endoscopic finding in children with *H. pylori* infection [34,37]. However, it could be caused by other viral infections (CMV, EBV), autoimmune disorders, NSAIDs use, or gastric cancer [2,36]. Nodular gastritis may be a good predictor of *H. pylori* infection [35,37], with high specificity (99.7%) but low sensitivity (7.2%) [38]. However, antral nodularity was still present in some *H. pylori*-negative patients (16.2%), possibly due to the spontaneous elimination of *H. pylori*, which can occur in around 2% of infected children [39,40,41]. 

PUD, though uncommon in children, can lead to severe complications such as GI bleeding or perforation [2]. This study recorded a high frequency of ulcers (19.4%), mainly located in the duodenum. Our results exceed the global rates reported in the clinical series, which ranged from 1% to 13% [30,42,43]. Notably, PUD in Vietnamese children seems to be on the rise or better recognized. The prevalence of PUD in children was only 1% in 2011 [11] and increased by 14,1% in 2014 [12]. More recently, in 2022, the ulcer rate was alarmingly high (30.8%) in Can Tho City, a neighboring province of HCMC [14]. Improved socioeconomic conditions and advancements in Vietnam’s health sector have made healthcare services more accessible [15]. Endoscopy indications in children have also been extended based on international guidelines [6]. Moreover, *H. pylori* are considered a major cause of PUD, especially in children [2,44,45]. Interestingly, in univariate analysis, PUD showed a significant increase with age and predominantly in boys. Prolonged *H. pylori* infection may lead to the development of PUD. The proportion of boys in the older age group (≥11 years) was higher than in other age groups. However, in multiple analysis, only gender was associated with PUD. The higher prevalence among boys could be attributed to a combination of genetic, hormonal, and immune responses.

Most *H. pylori* isolates carried the *cagA* gene (69%) and *vacA* s1/m1 genotype (44.3%). These strains are known to have a higher risk of developing PUD, as documented in the literature, including in Southeast Asian countries [46,47,48,49] and Vietnamese adults [50,51,52]. Interestingly, we detected mixed infections of *H. pylori* strains, with one-third of patients having coexistence of both *m1* and *m2* alleles of *vacA* (34.4% *s1/m1m2*), possibly because the genotype of *H. pylori* was studied directly from the gastric biopsy, rather than from a single colony. Multiple colonization with different *H. pylori* genotypes have been associated with gastritis features and ulceration [53]. These reasons may have contributed to the high frequency of PUD in symptomatic Vietnamese children requiring prompt diagnostic endoscopy.

Our study has three main limitations. Firstly, the number of patients was lower than expected due to recruitment interruption and disruptions in medical services, caused by the COVID-19 pandemic in Vietnam since February 2020. The adequate follow-up of patients was hampered by confinement. Secondly, such a low frequency of *H. pylori*-negative patients was not expected (20%). This may affect the study’s statistical power in identifying factors associated with *H. pylori* infection. Finally, some data remained subjective since the medical history and clinical symptoms were reported by parents of the children involved in the study. However, this study’s strength lies in being the first prospective multicenter design based on the international standard criteria for *H. pylori* diagnosis and endoscopic observation. We also excluded cases that may lead to an incorrect diagnosis, such as prior antibiotics, PPIs use, or repeated endoscopy, to limit selection bias. Therefore, our results accurately reflect the alarming frequency of *H. pylori* infection and PUD in symptomatic children in Southern Vietnam.

## 5. Conclusions

Vietnamese symptomatic children undergoing upper GI endoscopy have an alarming rate of *H. pylori* infection, up to 80%, from the age of four years. PUD is common in children in Southern Vietnam and is strongly associated with *H. pylori* infection. Health education programs focusing on the prevention and early detection of *H. pylori* should be established in Vietnamese children to mitigate the risk of ulcers and future gastric cancer.

## Figures and Tables

**Table 1:** Distribution of *H. pylori* infection according to characteristics of 336 symptomatic children.

Characteristics	*H. pylori*-Positive Children (n = 269)	*H. pylori*-Negative Children (n = 67)	*p*
Age group (years)—No (%)			0.96 *
<6	19 (7.1)	5 (7.5)	
6–11	190 (70.6)	48 (71.6)	
≥11	60 (22.3)	14 (20.9)	
Gender—No (%)			0.06
Girls	142 (52.8)	44 (65.7)	
Boys	127 (47.2)	23 (34.3)	
Living area—No (%)			<0.001
Other provinces	146 (54.3)	56 (83.6)	
- South-East provinces	71 (26.4)	23 (34.3)	
- Mekong Delta River	50 (18.6)	27 (48.2)	
- South Central Provinces	13 (8.9)	4 (7.1)	
Central Highlands	10 (6.9)	2 (3.6)	
- HCMC	123 (45.7)	11 (16.4)	0.90
- Rural	76 (61.8)	7 (63.6)	
- Urban	47 (38.2)	4 (36.4)	
Family history of gastric cancer—No (%)			>0.99
First-degree	0	0	
Second-degree	8 (3.0)	2 (3.0)	
No history	261 (97.0)	65 (97.0)	
Family history of PUD (n = 335)—No (%)			0.74
Yes	150 (56.0)	39 (58.2)	
No	118 (44.0)	28 (41.8)	
Family history of *H. pylori* infection—No (%)			0.21
Yes	135 (50.2)	27 (41.5)	
No	134 (49.8)	38 (58.5)	
Living with an infected person (n = 325)—No (%)			0.20
Yes	119 (45.4)	23 (36.5)	
No	143 (54.6)	40 (63.5)	
Nutritional status—No (%)			0.10
Normal weight	137 (50.9)	33 (49.3)	
Underweight	51 (19.0)	20 (29.9)	
Overweight/Obesity	81 (30.1)	14 (20.9)	
Signs and symptoms ^#^—No (%)			
Persistent abdominal pain	254 (94.4)	66 (98.5)	0.16
Epigastric pain	200 (74.4)	53 (79.1)	0.42
Vomiting	99 (36.8)	25 (37.3)	0.94
Constipation (n = 335)	90 (33.5)	27 (40.9)	0.26
Regurgitation	87 (32.3)	21 (31.3)	0.88
Weight loss	85 (31.6)	23 (34.3)	0.67
Loss of appetite	73 (27.1)	18 (26.9)	0.96
Heartburn	44 (16.4)	9 (13.4)	0.56
Dyspepsia	33 (12.3)	8 (11.9)	0.94
Anemia	32 (11.9)	8 (11.9)	0.99
Melena/Hematemesis	20 (7.4)	6 (9.0)	0.68
Diarrhea	20 (7.4)	6 (9.0)	0.68

* Cochran–Armitage test was used. ^#^ Children may have more than one symptom; So total percentage may exceed 100%. HCMC: Ho Chi Minh City. PUD: peptic ulcer disease.

**Table 2:** Association between endoscopic findings and *H. pylori* infection.

Endoscopic Findings	Positive *H. pylori* N = 269	Negative *H. pylori* N = 67	*p* **
Esophagus *			
Erosive oesophagitis	6 (100)	0 (0.0)	NA
Stomach and Duodenum			
Erythema	16 (6.0)	25 (37.3)	<0.001
- GEt	14 (5.2)	25 (37.3)	
- GEt & DEt	2 (0.8)	0 (0.0)	
Nodularity	171 (63.6)	33 (49.3)	0.03
- GN	69 (25.7)	9 (13.4)	
- GN & DEt	2 (0.8)	1 (1.5)	
- DN & GEt	6 (2.2)	2 (3.0)	
- DN & GN	94 (34.9)	21 (31.3)	
Erosion	21 (7.8)	5 (7.5)	0.93
- GEs	1 (0.4)	0 (0.0)	
- GEs & DEt	0 (0.0)	1 (1.5)	
- GEs & DN	1 (0.4)	0 (0.0)	
- DEs & DEt	1 (0.4)	3 (4.5)	
- DEs & GN	18 (6.7)	1 (1.5)	
Peptic ulcer disease	61 (22.7)	4 (6.0)	0.002
- GU & DN	1 (0.4)	0 (0.0)	
- DU	3 (1.1)	0 (0.0)	
- DU & GEt	3 (1.1)	0 (0.0)	
- DU & GN	50 (18.6)	4 (6.0)	
- DU & GEs	1 (0.4)	0 (0.0)	
- DU & GU	3 (1.1)	0 (0.0)	

* Patients may have simultaneous endoscopic lesions in the esophagus, stomach, and duodenum. ** Chi-square test on four categories. NA denotes not available. GEt: gastric erythema, GN: gastric nodularity, GEs: gastric erosion, GU: gastric ulcer. DEt: duodenal erythema, DN: duodenal nodularity, DEs: duodenal erosion, DU: duodenal ulcer.

**Table 3:** Logistic regression to assess factors associated with peptic ulcers among 269 *H. pylori*-positive children.

Characteristics	n	PUD (%)	Univariable Analysis OR (95% CI)	*p*	Multivariable Analysis OR (95% CI)	*p*
Age (years)				0.003		0.23
<11	209	18.7	1		1	
≥11	60	36.7	2.52 (1.34–4.74)		1.55 (0.76–3.14)	
Gender				0.003		0.04
Girls	142	15.5	1		1	
Boy	127	30.7	2.42 (1.34–4.36)		1.94 (1.02–3.66)	
Living area				0.14		0.26
- Other provinces	146	19.2	1		1	
- HCMC	123	26.8	1.55 (0.87–2.74)		1.43 (0.77–2.68)	
- Rural	76	25.0	1		NI	
- Urban	47	29.8	1.27 (0.56–2.87)	0.56		
Family history of PUD *				0.18		0.36
Yes	150	19.3	0.67 (0.38–1.20)		0.75 (0.40–1.40)	
No	118	26.3	1		1	
Nutritional status				0.68	NI	
Normal weight	137	24.8	1			
Underweight	51	19.6	0.74 (0.33–1.63)			
Overweight/Obesity	81	21.0	0.80 (0.42–1.56)			
Signs and symptoms						
Anemia				<0.001		0.13
- Yes	32	46.9	3.66 (1.70–7.87)		1.99 (0.81–4.87)	
- No	237	19.4	1		1	
Melena/Hematemesis				<0.001		0.07
- Yes	20	55.0	4.86 (1.91–12.4)		2.85 (0.92–8.86)	
- No	249	20.1	1		1	
*cagA* status *				<0.001		0.008
- Positive	185	29.2	4.48 (1.94–10.3)		3.25 (1.37–7.71)	
- Negative	83	8.4	1		1	
*vacA* genotypes **				<0.001		0.60
- *s1/m1*	86	31.4	1.83 (0.20–17.2)		0.74 (0.06–9.10)	
- *s1/m2*	106	11.3	0.51 (0.05–4.95)		0.28 (0.03–3.14)	
- *s2/m2*	4	0.0	1		1	
- *s1/m1m2* ^#^	52	40.4	2.71 (0.28–26.0)		1.62 (0.13–20.2)	
- *s1s2/m2* ^#^	5	20.0	NA		NA	
- *s1s2/m1m2* ^#^	2	0.0	NA		NA	
- *s2/m1m2* ^#^	1	0.0	NA		NA	
- incomplete *vacA* ^$^	8	0.0	NA		NA	

* One missing data. ** Five missing data. ^#^ Multiple *vacA* genotypes. ^$^ Incomplete *vacA* indicates only the s or m region was detected (7/8 *vacA s1* was detected, 1/8 *vacA m2* was detected). HCMC: Ho Chi Minh City. PUD: Peptic ulcer disease. NI: not included. NA denotes not available.

## Data Availability

The datasets generated and analyzed during the current study are available from the corresponding author upon reasonable request.
